# Severe Criticism

**Published:** 1920-06-12

**Authors:** 


### Severe Criticism.

### Health Ministry and Conditions in Nottingham.

The Minister of Health has sent a letter to the Nottingham Corporation refusing to grant powers to extend the boundaries of the city because the Council have "in some respects shown grave neglect in the discharge of their duties" under the Public Health Acts and the Housing Acts. The Minister says he is informed that about 30,000 pail closets are still in use in the city, and no satisfactory explanation has been given of the serious delay in improving upon this unhealthy and offensive system. The sewage-disposal arrangements are declared to be totally inadequate, and radical alterations are required to prevent the discharge of crude sewage into the river. The housing conditions are deplorable, and 10,000 workmen's dwellings are reported unfit for habitation; 7,000 of that number are described as incapable of being made fit, and the Council are themselves the owners of a number of houses condemned as unfit. Dr. Addison also expresses dissatisfaction with the progress made in carrying out the partial scheme submitted for the provision of new houses, and says he cannot grant any extension of boundaries until substantial improvements have been made in the sanitary and housing conditions within the existing boundaries.

The Council have hardly yet had time to reply to these severe criticisms—as no doubt they will—but in the meantime the *Nottingham Guardian* has devoted a leading article to the subject, which does not seem to present a very strong defence. It says the sanitary conditions are not worse than other cities that have extended their boundaries, but it asserts that for years it has been urging the Corporation to do what the Minister of Health now says ought to be done. The housing conditions "have long been a disgrace," and a menace to health. Pail closets were condemned years ago, and by very slow degrees are being got rid of, but the work was begun so long ago that it is difficult to remember the time. Agreement is expressed with the criticism of sewage disposal, and though Dr. Addison is said to have been a little hard upon the Corporation regarding the provision of houses, it is admitted that much more is needed; but attention is drawn to the difficulties, and it is held that Nottingham is building as fast as other places.

It is just as well that the Minister of Health should be perfectly blunt in impressing upon a Council its sanitary and public-health duties, and it is highly probable that his letter will make the whole subject one of first-class importance and debate in Nottingham. This is all to the good. Unfortunately it is probably true also that there are other cities open to just as much criticism as Nottingham has received, and for that reason it would be unjust to hold up Nottingham as an invidious and isolated example. But comparison with other cities just as bad, or worse, is not a defence to the Minister's grave charges if they are capable of being substantiated. There is an enormous amount of work to be done in public-health and sanitary arrangements, and one has a feeling that so much as been heard of the Ministry of Health, and so much hoped from it, that it is loosely imagined we are already marching vigorously on the high road, and ruthlessly sweeping aside all defects. It is a rough path yet before we get to the high road, even by the machinery of propulsion.

